# “Now I Don’t Have to Guess”: Using Pamphlets to Encourage Residents and Families/Friends to Engage in Advance Care Planning in Long-Term Care

**DOI:** 10.1177/2333721417747323

**Published:** 2017-12-20

**Authors:** Tamara Sussman, Sharon Kaasalainen, Matthew Bui, Noori Akhtar-Danesh, Susan Mintzberg, Patricia Strachan

**Affiliations:** 1 McGill University, Montreal, Quebec, Canada; 2 McMaster University, Hamilton, Ontario, Canada

**Keywords:** end of life, nursing homes, palliative care, advance care planning, comfort

## Abstract

**Objective:** This article explores whether access to illness trajectory pamphlets for five conditions with high prevalence in long-term care (LTC) can encourage residents and families/friends to openly engage in advance care planning (ACP) discussions with one another and with health providers. **Method:** In all, 57 residents and families/friends in LTC completed surveys and 56 participated in seven focus groups that explored whether the pamphlets supported ACP engagement. **Results:** Survey results suggested that access to pamphlets encouraged residents and families/friends to reflect on future care (48/57, 84%), clarified what questions to ask (40/57, 70%), and increased comfort in talking about end of life (EOL) care (36/57, 63%). Discussions between relatives and friends/families (32/57, 56%) or with health providers (21/57, 37%) were less common. Focus group deliberations illuminated that while reading illness-specific information was validating, a tendency to protect one another from an emotional topic, prevented residents and families/friends from conversing with one another about EOL issues. **Discussion:** Having access to pamphlets with information about EOL care provides important and welcome opportunities for reflection for both residents in LTC and their families/friends. Moving residents and families/friends from reflecting on issues to discussing them together could require staff support through planned care conferences or staff initiated conversations at the bedside.

## Background

Advance care planning (ACP) helps people with progressive, life-limiting illnesses reflect on and communicate values and preferences for future end-of-life (EOL) care to family, legally appointed decision-makers, and health providers (Jayaraman & Joseph, 2013; You et al., 2014). ACP is pertinent for residents in long-term care (LTC) because most are living with progressive, nonreversible, health conditions that make EOL planning real and near (Hirdes, Mitchell, Maxwell, & White, 2011). Furthermore, ACP fits with a person-centered approach, a perspective purported to guide practice in LTC, as it encourages residents to play a central role in directing their own EOL care while they are still able to do so (Kojima, 2015; Mitchell, Kiely, & Hamel, 2004).

Evidence is mounting on the positive impacts of ACP on EOL care for older adults in LTC (Berta, Laporte, Zarnett, Valdmanis, & Anderson, 2006; Hirdes et al., 2011; Mitchell et al., 2004; van der Steen, 2010; van der Steen, Radbruch, et al., 2014). Outcomes of ACP include more congruence between older adults’ wishes and care provided, lower rates of unnecessary hospitalizations at EOL, reduced stress, depression and anxiety among families, and improvements in care satisfaction for all parties (Berta et al., 2006; Brinkman-Stoppelenburg, Rietjens, & van der Heide, 2014; Detering, Hancock, Reade, & Silvester, 2010; Hirdes et al., 2011; Robinson et al., 2012; van der Steen, 2010).

Despite known benefits, ACP is rarely activated in LTC settings. Barriers include (a) staff discomfort introducing the topic of death or deterioration too early in the illness trajectory, (b) the concern that discussing death will reinforce the stigma associated with LTC (i.e., that of deterioration and neglect), (c) the lack of available tools to help direct discussions for conditions of high prevalence in LTC (e.g., dementia, heart failure), and (d) the belief that residents and families will not want to discuss death and dying in advance (Cable-Williams & Wilson, 2014; Mignani, Ingravallo, Mariani, & Chattat, 2017; Sussman et al., 2017).

In the absence of ACP, residents may be less likely to receive EOL care that is consistent with their preferences, should they become unable to articulate their wishes (You et al., 2014). Lack of ACP also places residents and families/friends at risk of distress, uncertainty, and dissatisfaction with EOL care provided (Brinkman-Stoppelenburg et al., 2014; Wright et al., 2008).

Addressing these noted barriers to ACP in LTC, our team developed, implemented, and evaluated the effects of disease-specific pamphlets for five life-limiting advanced conditions of high prevalence in LTC: dementia, heart failure, chronic obstructive pulmonary disease (COPD), renal failure, and frailty (Hirdes et al., 2011). Our team iteratively developed the five 8 × 11 threefold pamphlets based on current evidence, patient education literature, and the team’s collective expertise in ACP, palliative care, and EOL care. The final versions of the pamphlets included the following topics: (a) the relevance of ACP, (b) a description of the specific condition, (c) signs and symptoms of advanced stages of the illness, (d) tips for caregivers, and (e) links to online resources (see the following website to view the pamphlets: http://www.chpca.net/projects-and-advocacy/projects/strengthening-a-palliative-approach-in-long-term-care-spa-ltc-project.aspx).

Face and content validity were achieved prior to pamphlet distribution and evaluation based on consultations with palliative care specialists and palliative care leaders within the four LTC sites where this study was conducted (see— Strachan, Bui, Durepos, Sussman, & Kaasalainen, 2016; Sussman, Bui, Kaasalainen, Venturato, & SPA-LTC Team, 2016—for further details regarding pamphlet development).

Part of a larger study called Strengthening a Palliative Approach to Care in Long-Term Care (SPA-LTC), this article is based on survey responses and focus group deliberations with residents and families/friends who used the pamphlets. Both surveys and focus groups aimed to (a) explore reactions to receiving information about ACP through the pamphlets, (b) identify the extent to which receiving the pamphlets activated engagement in ACP, (c) examine differences between residents’ and families/friends’ reactions and perceived ACP engagement, and (d) inform when and how the pamphlets should be distributed in LTC.

## Method

This study used a mixed methods design that included both a survey (quantitative) and a focus group (qualitative) component. First, survey data was collected from residents and families/friends who used the pamphlets in four participating nursing homes in southern Ontario to explore (a) overall reactions to pamphlet use, (b) perceived engagement with ACP a result of pamphlet use, and (c) differences between residents and families. Second, focus groups were conducted with residents and families/friends who had seen and read the pamphlets to gain further insight into (a) overall reactions to the pamphlets, (b) how and why the pamphlets may or may not have resulted in ACP engagement, and (c) considerations for future implementation.

The research was conducted in accordance with the standards of the Tri-Council Policy Statement for Ethical Conduct for Research Involving Humans 1998 (with 2000, 2002, and 2005 amendments). Procedures were approved by the Office of Research Ethics Board at McGill University and McMaster University.

### Surveys

#### Recruitment and sampling

Over a period of 6 months (March-August 2016), pamphlets were made available to residents and families/friends in the four LTC homes through bulletin displays or through distribution by staff at care planning meetings or informal bedside discussions. Anonymous paper-based surveys were available in bulletin board displays for those who took the pamphlets themselves or distributed along with the pamphlets for those who received the information from staff.

A total of 348 pamphlets were either taken or distributed over the course of the study. Although 57 residents and families/friends completed and returned surveys, we cannot report a response rate as we do not know how many residents or families/friends either took or received a pamphlet.

#### Measures

In accordance with the health behavior literature, the research team developed a 19-item survey to evaluate the pamphlets. The survey aimed to capture reactions to the pamphlets (i.e., how the pamphlets were used, their perceived usefulness, their linguistic accessibility, negative reactions) and to assess ACP engagement (i.e., improved comfort, supported action; Agency for Healthcare Research and Quality, 2015; Gruman et al., 2010; Koh, Brach, Harris, & Parchman, 2013; Osbourne, 2001; Rizzo et al., 2010). More specifically, the survey captured the following:

Reactions:
1. *Pamphlet use*: captured by two categorical questions asking participants to identify the selected pamphlet(s) and the part(s) of the pamphlets read.
2. *Information accessibility*: captured by two questions asking participants to rate the extent to which the information was clear and easy to understand.
3. *Perceived Usefulness*: captured by three questions including one categorical question asking respondents to report on sections of the pamphlets they found useful and two questions asking participants to rate the extent to which the pamphlet was helpful and meaningful/relevant.
4. *Negative reactions*: captured by two questions asking participants to rate the extent to which the pamphlet caused feelings of distress or the degree to which sections of the pamphlet were perceived as unhelpful.
ACP engagement:
5. *Comfort with ACP conversations*: captured by six questions asking about improved comfort and intentions to activate conversations as a result of using the pamphlets.
6. *Activation of ACP conversations*: captured by four questions asking about actual conversations initiated as a result of using the pamphlets.

Respondents were asked to rate items in Domains 2 to 6 on a 5-point Likert-type scale ranging from *strongly agree* (1) to *strongly disagree* (5). Demographic characteristics including gender identity, education, primary language spoken, and respondent type (resident or family/friend) were captured to describe the sample and allow for comparisons of overall use and perceptions.

### Method of Analysis

Descriptive statistics (means and standard deviations for continuous data and proportions and percentages for categorical data) were conducted to provide an overview of sample characteristics, overall use, and reactions to the pamphlets. For descriptive purposes, strongly agree and agree responses were grouped together to represent agreement for an associated item.

An exploratory factor analysis using principal components analysis and varimax (orthogonal) rotation was conducted for the 17 Likert-type scale items on the survey to examine if survey items clustered around preconceived domains (Pett, Lackey, & Sullivan, 2003). This analysis allowed us to conduct Student’s *t* tests comparing means for reactions and ACP engagement by role (resident vs. family/friend), education level (high school vs. post-secondary), and method of distribution (taken by self or received by others). Differences were also compared using chi-square analyses for categorical variables.

We examined education level because it is often considered an important element of health behavior (Sabharwal, Badarudeen, & Kunju, 2008; van der Steen, Radbruch, et al., 2014). Forms of distribution were also compared because there has been much debate in the literature regarding when and how ACP should be activated (van der Steen, van Soest-Poortvliet, et al., 2014). All statistical analyses were conducted using SPSS version 23.0 statistical software.

### Focus Groups

#### Recruitment and sampling

All four partnering LTC homes were asked to recruit participants for two distinct focus groups: residents and families/friends of current residents. All families/friends and residents who staff deemed to have functional capacity to take part in a discussion-based focus group, and who had indicated that they had seen and read the pamphlets, were eligible to participate. LTC staff in participating homes used the following recruitment strategies to invite potential participation: emails, flyers, and signup sheets within the homes.

A total of 56 people agreed to participate. This included 36 residents participating in four focus groups (one at each participating site) and 20 family members/friends participating in three focus groups (one LTC home site could not convene a focus group of families/friends). Because recruitment was led by LTC staff, those who may have declined participation were not recorded.

#### Measures

All focus groups were conducted in the summer of 2016 and were facilitated by two members of the research team (one of whom asked questions and the other who took field notes). A semistructured interview guide was developed to guide focus group discussions. The guide probed participants’ perceptions of the information provided in the pamphlets; ideas to guide implementation and the extent to which the pamphlets supported comfort initiating ACP. Each focus group was tape recorded and then transcribed verbatim. Field notes were also taken during the focus groups capturing main themes emerging from the discussion, the facilitators’ impressions of the group dynamic, the level of participation, and any other observations related to the overall discussion. Participants were also asked to complete a short questionnaire which asked about gender identity, age, and length of time in LTC. The focus groups ranged in duration from 25 min to 45 min.

#### Method of analysis

A conventional three-part content analysis was performed by two members of the research team (TS & SM) (Hsieh & Shannon, 2005). In the first stage, all text segments were assigned preliminary categories based on the broad areas probed in the interview guide. Preliminary categories at this stage included pamphlet accessibility, usefulness/relevance, actions taken/considered, and recommendations for implementation. In the second stage, two team members (TS & SM) reviewed the coded data looking for differences and similarities within and across categories. At this stage, preliminary categories were combined and some were reconsidered. For example, while pamphlet accessibility was retained as a category at this second stage, text segments initially coded under recommendations for implementation were reconsidered as either related to accessibility or an underlying level of comfort/discomfort around initiating ACP conversations with relatives. In the third and final stage, all coded data were reexamined by two members of the research team (TS & SM)) independently. They then analyzed together how the preliminary categories expanded, contradicted, or affirmed trends noted in the quantitative data. At this final stage, three themes emerged: *pamphlet (in)accessibility, the value of illness-specific information*, and *protecting one another from an emotional topic:* *a barrier to pamphlet use*. These themes represented ideas discussed extensively within and across groups, and appeared to shed light on some of the trends noted in the quantitative data. These three themes and coded excerpts were reviewed by other members of the research team who agreed that they were comprehensive and reliably represented comments made by participants.

## Results

### Survey Results

#### Characteristics of the sample

Table 1 provides an overview of participants who completed the surveys. Participants were primarily female with a mean age of 65.8 years (*SD* = 15.2). Thirty-six out of 57 participants (63.2%) were LTC residents. Of the 21 family members/friends who completed the survey, approximately half were adult children. More than half of participants completed high school, and all spoke English as their first language. More residents reported ceasing their education at the high school level than family/friends.

**Table 1.** Overview of Resident and Family/Friend Characteristics and Pamphlet Use.

Characteristic	Total sample, *n* (%) (*N* = 57)	Resident, *n* (%) (*n* = 36)	Family/friend, *n* (%) (*n* = 21)
Age, *M* (*SD*)	65.8 (15.2)	69.9 (11.5)	59.0 (18.3)
Gender, *n* (%)			
Male	16 (28.1)	14 (38.9)	2 (9.5)
Female	40 (70.2)	21 (58.3)	19 (90.5)
Relationship to resident, *n* (%)			
Husband/wife	—	—	5 (23.8)
Son/daughter	—	—	11 (52.4)
Other relative	—	—	2 (9.5)
Other (i.e., friend)	—	—	3 (14.3)
Education level, *n* (%)			
High school	32 (60.4)	23 (71.9)	9 (42.9)
Postsecondary	21 (39.6)	9 (28.1)	12 (57.1)
Primary language, *n* (%)			
English	56 (98.2)	35 (97.2)	21 (100)
Method of pamphlet distribution			
By self	29 (50.9)	15 (41.7)	14 (66.7)
By staff	28 (49.1)	21 (58.3)	7 (33.3)
Percentage of sections read, *n* (%)			
≥50% of sections	47 (83.9)	30 (85.7)	17 (81.0)
Sections read, *n* (%)			
Disease definition	43 (75.4)	27 (75.0)	16 (76.2)
Signs/symptoms	46 (80.7)	30 (83.3)	16 (76.2)
Tips for caregivers	41 (71.9)	25 (69.4)	16 (76.2)
Palliative approach	47 (82.5)	29 (80.6)	18 (85.7)
Resources	48 (84.2)	31 (86.1)	17 (81.0)
Percentage of sections found useful, *n* (%)			
≥50% of sections	39 (68.4)	24 (66.7)	15 (71.4)
Sections found useful, *n* (%)			
Disease definition	38 (66.7)	25 (69.4)	13 (61.9)
Signs/symptoms	38 (66.7)	25 (69.4)	13 (61.9)
Tips for caregivers	37 (64.9)	20 (55.6)	17 (81.0)
Palliative approach	38 (66.7)	22 (61.1)	16 (76.2)
Resources	36 (63.2)	24 (66.7)	12 (57.1)
Number of pamphlets read per person			
One pamphlet	38(66.7)	28 (77.8)	10 (47.6)
Two pamphlets	7 (12.3)	3 (8.3)	4 (19.1)
Three of more	12 (21)	5 (13.9)	7 (33.3)
Pamphlets read, *n* (%)			
Heart failure	27 (47.4)	17 (47.2)	10 (47.6)
Advanced dementia	21 (36.8)	10 (27.8)	11 (52.4)
COPD	10 (17.5)	7 (19.4)	3 (14.3)
Advanced renal disease	14 (24.6)	8 (22.2)	6 (28.6)
Frailty	28 (49.1)	13 (36.1)	15 (71.4)**

*Note.* Total percentages may not equal 100% due to missing responses and/or because some responses to pamphlet sections are not mutually exclusive. COPD = chronic obstructive pulmonary disease.

** Significance < .01.

#### Pamphlet distribution and use

Table 1 provides an overview of how pamphlets were received, the extent to which they were read, and any differences between families/friends and residents on distribution and use. Fifty-one percent of participants (29/57) reported having taken the pamphlet from a bulletin board and 49% of participants (28/57) reported having received it from a staff member. Forty-two percent of residents (15/36) took the pamphlets themselves while 58% (21/36) were given the pamphlets by someone in LTC. By contrast, 67% (14/21) families/friends took the pamphlets themselves and 33% (7/21) received the pamphlets from someone in LTC. Thirty-three percent of participants (19/57) reported receiving or taking more than one pamphlet. On average, residents and families/friends took or received two pamphlets each (details not show in table).

Illness trajectory pamphlets for heart failure (27/57, 47.4%), frailty (28/57, 49.1%), and dementia (21/57, 36.8%) were read with the highest frequencies. More families/friends read the frailty pamphlets than residents.

More than 80% of participants (47/57) read more than half of the pamphlets and nearly 70% of participants (39/57) found more than half of the sections of the pamphlets to be useful. The relevance of ACP (47/57, 72.5%) and signs/symptoms of advanced illness (46/57, 80.7%) were the most frequently read parts of the pamphlets. Most participants found all sections read to be useful with slightly higher endorsement for the relevance of ACP, disease definition, and signs and symptoms sections. No significant differences were found between residents and families/friends when examining the percentage of sections read, amount of pamphlets reviewed, or sections that were found to be useful.

#### Survey items associated with reactions and engagement with ACP

Table 2 presents the results of our factor analysis. The initial 17-item Likert-type scale component of our survey was designed to capture two domains: overall reactions to the pamphlets (usefulness, accessibility, and negative reactions) and perceived engagement with ACP (ACP comfort and ACP activation). However, the factor analysis revealed three factors with eigenvalues greater than 1. Examination of item loadings led the team to reconceptualize items as representing the following three categories: (a) positive reactions (five items, Cronbach’s alpha .87), (b) negative reactions (two items, Cronbach’s alpha .66), and (c) overall engagement with ACP (eight items related to comfort and action, Cronbach’s alpha .91). Two items considered to capture ACP engagement were dropped for the purposes of mean comparisons (“I was encouraged to think about my [or my family/friend’s] values or goals of care” and “I know what to ask about future care needs”) because they were loaded onto conceptually inappropriate factors. The final 15 Likert-type scale items explained 69.78% of the variance and had primary loadings of over 0.65. Our dichotomous groupings of overall reactions and engagement in ACP revealed the following results (not presented in a table).

**Table 2.** Survey Items Associated With Reactions and Engagement With ACP.

Item	Positive reactions	Engagement of ACP	Negative reactions	*M* (*SD*)
The information in this pamphlet was presented clearly.		0.8880		4.47 (0.87)
The information in this pamphlet was easy to understand.		0.8747		4.52 (0.67)
The information in this pamphlet was helpful.		0.8512		4.33 (0.66)
The information in this pamphlet was meaningful and relevant.		0.8955		4.19 (0.68)
The information in this pamphlet was upsetting or distressing.			0.8933	1.95 (1.20)
The information in this pamphlet provided had sections that I felt were not important.			0.8216	2.67 (1.28)
The information in this pamphlet provided very helpful online resources.		0.6568		4.25 (0.72)
I feel more comfortable to explore my (or my family or friend’s) values and preferences about palliative/end-of-life (EOL) care.	0.7014			3.95 (0.69)
I want to speak with a health care provider about the information in this pamphlet.	0.7381			3.80 (0.95)
I feel more knowledgeable about the trajectory of my illness (or the illness of my family member or friend).	0.7405			3.56 (1.10)
I intend to share this information with a family member or friend.	0.8133			4.21 (0.79)
I started to speak to my family member(s) or friend about care preferences or values.	0.7612			4.05 (0.69)
I have spoken with a health care provider about some or all of the information in this pamphlet.	0.7928			3.00 (1.33)
I plan to have more conversations with my (or my family member or friend’s) health care team about care preferences and values in the future.	0.8328			4.05 (0.89)
I plan to have more conversations with my family member(s) or friend about care preferences and values in the future.	0.8380			4.15 (0.81)

*Note.* The following items did not conceptually fit into their loaded factor and were subsequently removed from the factor analysis: “I was encouraged to think about my (or my family or friend’s) values or goals of care” and “I know what to ask about future care needs.” ACP = advance care planning.

##### Positive reactions

Most respondents found the content clear (47/57, 82%), easy to understand (50/57, 88%), helpful (45/57, 79%), and meaningful and relevant (47, 82%).

##### Negative reactions

Few participants found the information contained in the pamphlets distressing (11/57, 19%) or nonrelevant (15/57, 26%).

##### Engagement with ACP

Overall, participants reported feeling encouraged to think about personal values and goals of care (48/57, 84%) and more informed about what to ask regarding future care needs (40/57, 70%) after reading the pamphlets. Although many participants reported an intention to share this information with families/friends (40/57, 70%) and an increased comfort talking about EOL care after reading the pamphlets (36/57, 63%), fewer had started speaking with families/friends (32/57, 56%) or health providers (21/57, 37%) about these issues after reading the pamphlets. Fewer also reported a desire to speak to a health provider within LTC about the information in the pamphlets (25/57, 44%).

#### Comparisons

Table 3 shows comparisons of positive reactions, negative reactions, and ACP engagement between residents and families/friends, by method of distribution, and by educational attainment. People with higher educational attainment tended to report more positive reactions and less negative reactions than persons with lower attained educational levels. There were no significant differences in activation of ACP, negative reactions, or positive reactions between residents and families/friends, or by method of distribution.

**Table 3.** Comparisons of Positive Reactions, ACP Engagement, and Negative Reactions Between Residents and Families/Friends, Method of Distribution, and Education Level.

Demographic variable	Positive reactions (5 items)		Engagement of ACP (8 items)		Negative reactions (2 items)		Total score (15 items)	
	*M* (*SD*)	*p* value	*M* (*SD*)	*p* value	*M* (*SD*)	*p* value	*M* (*SD*)	*p* value
Role		*.060*		.287		.283		.224
Resident	3.87 (0.8)		3.55 (0.9)		2.65 (1.2)		3.54 (0.6)	
Family/friend	4.26 (0.6)		3.80 (0.8)		2.31 (1.0)		3.74 (0.6)	
Education		*.004*		.916		*.019*		.537
High school	3.82 (0.7)		3.62 (0.9)		2.73 (1.2)		3.56 (0.6)	
Postsecondary	4.40 (0.6)		3.59 (0.7)		1.97 (0.9)		3.66 (0.6)	
Method of distribution		.955		.097		.717		.145
By self	4.02 (0.6)		3.46 (0.8)		2.47 (1.0)		3.50 (0.6)	
By staff	4.01 (0.9)		3.83 (0.8)		2.58 (1.3)		3.73 (0.6)	

*Note. p* values ≤ .05 are italicized. ACP = advance care planning.

### Focus Group Results

#### Characteristics of the sample

Focus group participants were predominantly female (41/56, 73%), ranged in age from 35 to 86, and had been living with or supporting a relative in LTC for at least 1 year. Just under half of the family members/friends were adult children (9/20, 45%) with the remainder evenly distributed between spouses, siblings, and other friends/relatives.

#### Themes

Analysis of the focus group deliberations revealed three themes that provide further insights into residents’ and families/friends’ use of and reactions to the pamphlets as a mechanism for activating ACP in LTC. Together the three themes, *Pamphlet (in)accessibility; The value of illness-specific information*; and *Protecting the other from an emotional topic: A barrier to pamphlet use*, offer important considerations for future implementation.

##### Pamphlet (in)accessibility

Participants in all focus groups suggested that the pamphlets were helpful in supporting an understanding of a palliative approach to care and in describing some of the most relevant aspects of planning for EOL care for various illnesses. Both residents and families/friends suggested the information was clear and the language was accessible which was considered important to support use. A resident commenting on the concise nature of the information stated, “It tells you a lot of what you need to know without being too wordy and making you read through a lot of things” (Resident Participant, Site 1). A family member commenting on accessibility said, “Yeah I like the fact that it’s plain language not medical terminology. Anybody can read it and get something out of it without running for a dictionary” (Family Participant, Site 4). Both residents and families agreed that the pamphlets were accessible in that they provided useful information which was easy to understand.

Importantly, one element of accessibility emerging from focus group discussions was the location of the pamphlets within the homes. Residents noted that placing the pamphlets in a display board at the front of the home only, made them more visible and accessible to families/friends than to residents. Suggestions for better accessibility included lowering the racks that contained the pamphlets so that people in wheelchairs could see and access them, placing racks with pamphlets on every floor in the residence, and having racks with pamphlets in high traffic areas such as near elevators, nursing stations, lobbies, and common rooms, such as a TV lounge.

##### The value of illness-specific information

While most focus group participants had been conceivably living with or supporting someone with a chronic condition for several years, many suggested that the pamphlets provided new information, prepared them for what to expect and what to look out for, and validated what they had been experiencing and/or observing. As one family member explained,

When I saw these pamphlets and started reading, I went oh my gosh, okay this is exactly him right now this is what I have to expect, this is where I can look for help and all of sudden you don’t feel helpless, you feel like this is normal, this is what, you know . . . this is what is supposed to be happening. (Family Participant, Site 2)

Another family member supporting a relative with dementia for many years stated,

I just I felt like I knew what I could ask and I knew what I had to look for and what needed to be done for him because honestly I didn’t have a clue with dementia, so this honestly it really helped. (Family Participant, Site 2)

Similar sentiments were expressed by residents who emphasized the value of being well informed,

Well I like the, in the pamphlet I’m looking at is the advanced kidney disease and um the inside section on “what is advance kidney disease?” and here it outlines very precisely is what it is and what the effects are going to be and I like that very much because I didn’t really know all of this and I find looking that these pamphlets, for me, it’s quite a learning experience. (Resident Participant, Site 1)

Another resident emphasizing the value in learning more about a particular illness stated, “Knowledge is a good thing and it, it’s a doorway to finding out more if you want to or need to” (Resident Participant, Site 1).

For some, seeing the information in print helped them to confirm their own impressions of where in the illness trajectory they/or their relatives were. As a family member stated, “Yeah it just confirms what you’ve been seeing. It’s good to see it confirmed because you wonder if that’s part of the process” (Family Participant, Site 1).

Although disease definitions and illness trajectories did not stand out as significantly more helpful than other components of the pamphlet in the surveys, focus groups affirmed the particular benefits of these elements of the pamphlets suggesting the information was welcome, empowering, and validating. As one family member stated,

I think the other encouraging thing or positive thing on my end, looking, is the actual symptoms of whatever your person or loved one is dealing with because you might look at a symptom and think oh it’s related to something else when it’s actually a progression of whatever they have. So, this can help the families be better prepared. (Family Participant, Site 4)

##### Protecting the other from an emotional topic: A barrier to pamphlet use

Participants expressed differing opinions on when pamphlets should be distributed with some suggesting they should be given out on admission to LTC and others noting the importance of allowing people to adjust to LTC prior to being given such information. Further analyses of this discrepancy suggested that in general, both resident and family/friend participants felt that *they* would like the information earlier but wondered if *the other* would be ready for it. Common responses to their own preferences for the timing of receiving the pamphlets residents and families/friends were the following: “I like the idea of the early start, like integrating it right away” (Family Participant, Site 4) and “Oh yes, you should know everything about it. Nothing should be held back” (Resident Participant, Site 3). However, when referring to how the other party (families/friends for residents and residents for families/friends) would feel about receiving the information early, they expressed more apprehension. One family member speaking of resident readiness stated, “This is something like, you know like you say, you get somebody in here that doesn’t want to be here you know they don’t want to see this right away” (Family Participant, Site 2). A resident speaking of a family member’s readiness for information said, “Some family members they don’t want it, some family members do not want to hear about all this stuff but they need to” (Resident Participant, Site 2).

For the most part, concerns and apprehensions about other’s reactions revolved around their anticipated emotional reactions. A family member explaining why she felt uncomfortable with the idea of her husband receiving such a pamphlet stated, “Because if he thought he was dying, I don’t know if he would like to know that, and he might just give up” (Family Participant, Site 1). A resident, who expressed apprehension about her daughter receiving the pamphlets was asked by the facilitator what she thought would happen, if her daughter read the pamphlets to which she replied, “I think she’d be upset” (Resident Participant, Site 3).

Interestingly, despite this sense of family members and residents protecting each other from the information that they themselves found helpful, participants who had previously had open conversations within their families indicated a sense of relief at having opportunities to discuss these issues together. One family participant who was invited to a care conference with her relative to discuss EOL care wishes stated,

She was very detailed about how she wants it all to go and play out. I couldn’t have been more surprised. And it makes it easier for me right, because now I don’t have to guess. So now I know that when it gets to that time, and I’m not thinking clearly anyways I won’t have to guess. (Family Participant, Site 4)

Another family participant explained that following the staff’s initiative, she and her mother were able to have a conversation about her mother’s EOL wishes. She states,

I wouldn’t have known if I wasn’t asked. Like I was literally asked, would you and your mom like to do this now? So, I asked my mom and she was all over it, like yes I would. But left to our own devices I don’t think it would have happened because we didn’t know. (Family Participant, Site 4)

Taken together, these findings suggest that while residents and families welcome information about disease progression, they each worry about how the other will react to such information. This could pose a barrier to moving from raised awareness to actively engaging in conversations with the other about EOL issues. Those who had opportunities to discuss care preferences and wishes with their family members did so in the context of facilitated conversations with staff. They appreciated these opportunities finding them surprisingly reassuring.

## Discussion

Overall, our study results suggested that illness trajectory pamphlets were an acceptable way of offering information to residents and families/friends about what to expect and ask about regarding future care issues for illnesses common to residents in LTC. After taking or receiving a pamphlet, many participants reported being more informed and prepared for what to ask about and discuss and few noted negative effects. This suggests that illness trajectory pamphlets may offer a mechanism for priming and preparing both residents and families/friends to participate in conversations with one another about ACP and related preferences, fears, and concerns (Sudore et al., 2008). Notably, residents were less likely than families/friends to read the frailty pamphlets which may represent failure to self-identify with a term associated with negative connotations such as decline and weakness (Grenier, 2007). Also notable was the tendency for residents and families/friends to take or receive multiple pamphlets. This may suggest that many respondents were concerned about multiple conditions and needed to consult with more than one pamphlet (Mercer, Smith, Wyke, O’Dowd, & Watt, 2009; Van Cleave et al., 2016).

Residents and families/friends reported greater interest in discussing EOL issues with one another than with health providers in LTC. Although ACP continues to be defined as a process that supports communication between patients, families/friends, and health providers, much of the literature informing ACP has focused on activating communication between *patients* and *health providers* (typically physicians) with the aim of fostering patient autonomy and recording goals of care (Brinkman-Stoppelenburg et al., 2014). Our findings suggest that within the context of LTC, an important and pressing component of ACP for both residents and families/friends is that of communication *within* families. This finding lends some support to the model put forth by Sudore and colleagues (2008) who conceptualize ACP as a series of sequential steps and consider discussions *within* families to be an important precursor to discussions with clinicians about goals of care (McMahan, Knight, Fried, & Sudore, 2013; Sudore et al., 2008).

Our combined survey and focus group data also suggested some notes of caution regarding the capacity for educational pamphlets to activate and encourage communication between residents, families, and staff regarding EOL issues. First, residents and families/friends with higher education found the information included in the pamphlets to be more relevant and accessible, and reported fewer negative reactions than those with lower levels of education. This suggests that illness trajectory pamphlets that aim to activate and prime older adults and families/friends to think about ACP may be particularly appropriate for health literate segments of the LTC population. Others have similarly found that older adults with higher levels of education appear to benefit more from health education initiatives than older adults with lower educational levels (Cutilli, 2007; Hsieh & Shannon, 2005).

Second, although methods of pamphlet distribution did not affect reactions, or activation of ACP, families/friends were more likely to take the pamphlets themselves while residents were more likely to receive the information from staff. Focus group deliberations suggested that this difference may have been related to where the pamphlets were situated within the LTC home, which is at the entrance of the homes where families/friends often passed and residents rarely convened. To improve accessibility for residents, pamphlets should be made visible and available in parts of the home typically frequented by the residents. Engaging residents in discussions regarding where such information should be located could be an important step in ensuring equitable access of this type of information for residents. It could also help to alert residents to the availability of such information should they be interested. Ensuring residents and families/friends both have access to information about potential signs and symptoms of deterioration is imperative, if the goal includes empowering residents to become active participants in their own EOL planning (Arcand et al., 2013; Arcand et al., 2009).

Third, and perhaps most importantly, although residents and families/friends alike indicated an interest in discussing EOL issues with one another, receiving or taking the pamphlets did not, for the most part, activate discussions between residents and their families/friends. Focus group deliberations suggested that this additional step may require further encouragement by staff to help relieve deep-seated fears and concerns about how “the other” may react to such conversations. Knowing what to discuss is only one step toward activating ACP between residents and families/friends. Both need to be prepared and equipped to manage and to tolerate the emotional reactions of the other. Schickedanz and colleagues (2009) suggested that major barriers for activating discussions within families about EOL issues included patients’ concerns about family burden, lack of family, or poor relational dynamics. Our findings suggest that concerns about emotionally burdening the other extends beyond patients themselves and also interferes with families/friends’ capacity to activate discussions with residents even when such discussions are considered important and desirable.

Focus group deliberations also suggested the value and relief afforded to families who had the opportunity to engage in such discussions with residents. These conversations were typically invited by staff either within the context of formalized care conferences or informally at the bedside. Reaching out and providing residents and families with the opportunity to express emotion in a contained environment appears to be important steps to move residents and families from contemplation to action about ACP in a LTC home environment (Dev et al., 2013). This suggests that some form of staff follow-up is warranted following pamphlet distribution. Such conversations could be initiated by simply reasking families and residents to discuss with one another what the pamphlets made them think about and to offer them opportunities to openly and honestly discuss concerns, fears, and wishes with one another.

The provision of staff support for EOL conversations requires a level of staff comfort to invite and facilitate such conversations between residents and families. However, evidence suggests that this level of staff comfort cannot be presumed in the current LTC context (Arcand et al., 2013; Arcand et al., 2009; Sussman et al., 2017). Hence, staff training may be another necessary component for supporting successful implementation.

Much of the literature on staff-facilitated ACP in LTC includes a component that encourages staff documentation of ACP discussions (Brinkman-Stoppelenburg et al., 2014; Cornally et al., 2015; Sinclair, Oyebode, & Owens, 2016). Our collective findings suggest that prior to such formalized documentation, residents and families/friends need many more opportunities to discuss their fears, concerns, and preferences for EOL care with one another (Lintzelman et al., 2017; Sudore & Fried, 2010; Sudore et al., 2008).

## Study Limitations

First, our factor analysis can only be considered exploratory because our sample size was small. However, our results may be useful in future studies aimed at the development of standardized evaluation measures for patient and family educational material. Second, all participants reported English as their first language. Given that linguistic differences regarding the acceptability of ACP and shared information about EOL symptoms has been documented, future work should examine acceptability of similar pamphlets among individuals whose mother tongue is not English (Arcand et al., 2013). Third, resident and family/friend perceptions captured in this study were based on a small self-selected sample whose experiences may not be transferable to other residents and families/friends in LTC. Finally, the survey data asked individuals to self-report activation of ACP conversations. It is possible that individuals overreported their engagement in actual conversations with family/friends as a result of reading the pamphlets. Our focus group deliberations lent some support to this possibility by identifying the reservations that many participants had moving from thinking to talking about EOL issues with one another.
